# EXAMINING THE DYNAMIC PROCESS LINKING PERSONALITY, HEALTH BEHAVIORS AND STRESS, AND HEALTH OUTCOMES

**DOI:** 10.1093/geroni/igae098.0553

**Published:** 2024-12-31

**Authors:** Jing Luo, Bo Zhang, Tomiko Yoneda, Eileen Graham, Daniel Mroczek

**Affiliations:** Northwestern University, Evanston, Illinois, United States; University of Illinois Urbana-Champaign, Champaign, Illinois, United States; University of California Davis, Davis, California, United States; Northwestern University, Evanston, Illinois, United States; Northwestern University, Evanston, Illinois, United States

## Abstract

Personality has been found to contribute to individual differences in health and aging. In search of the underlying mechanisms, health behavior and stress have been shown to play roles in the personality-health association. However, given the evidence that personality traits are dynamic within individuals, it remains unclear how the dynamic intraindividual connections between personality and health behavior engagement and stress experiences contribute to interindividual differences in health and health change over time. Using 10-wave data from the Longitudinal Internet Studies for the Social Sciences (N = 3,421), the current study examined the effects of the intraindividual interplays between changes in personality and changes in health behaviors and stress on individual differences in different health outcomes and changes in health outcomes. Adopting multilevel structural equation modeling, the results suggested that after controlling personality at the interindividual level, the dynamic intraindividual interplays between extraversion, openness and smoking at each time point still displayed significant effects on interindividual differences in smoking, which in turn significantly contributed to interindividual differences in self-rated health and symptom complains in the last wave and changes in general disease level and cardiovascular diseases over time. Meanwhile, the dynamic intraindividual interplays between extraversion, conscientiousness and perceived stress were significantly related interindividual differences in perceived stress, which were significantly linked to interindividual differences in the levels of and changes in all health outcomes. The current findings enhance our understanding of how the dynamic intraindividual connections between personality and people’s behaviors and experiences may inform interindividual differences in health during aging.

